# State-of-the-Art Molecular Oncology of Lung Cancer in Taiwan

**DOI:** 10.3390/ijms23137037

**Published:** 2022-06-24

**Authors:** Yung-Hung Luo, Kung-Hao Liang, Hsu-Ching Huang, Chia-I Shen, Chi-Lu Chiang, Mong-Lien Wang, Shih-Hwa Chiou, Yuh-Min Chen

**Affiliations:** 1 Department of Chest Medicine, Taipei Veterans General Hospital, Taipei 11217, Taiwan; hecterlo@gmail.com (Y.-H.L.); hchuang15@vghtpe.gov.tw (H.-C.H.); cishen2@vghtpe.gov.tw (C.-I.S.); clchiang@vghtpe.gov.tw (C.-L.C.); 2 School of Medicine, National Yang Ming Chiao Tung University, Taipei 11221, Taiwan; monglien@gmail.com; 3 Department of Medical Research, Taipei Veterans General Hospital, Taipei 11217, Taiwan; kunghao@gmail.com; 4 Institute of Food Safety and Health Risk Assessment, National Yang Ming Chiao Tung University, Taipei 11221, Taiwan; 5 Institute of Biomedical Informatics, National Yang Ming Chiao Tung University, Taipei 11221, Taiwan; 6 Institute of Clinical Medicine, National Yang Ming Chiao Tung University, Taipei 11221, Taiwan; 7 Institute of Pharmacology, National Yang Ming Chiao Tung University, Taipei 11221, Taiwan

**Keywords:** Taiwan, lung cancer, precision medicine, proteogenomics, targeted therapy, NRF2

## Abstract

Lung cancers are life-threatening malignancies that cause great healthcare burdens in Taiwan and worldwide. The 5-year survival rate for Taiwanese patients with lung cancer is approximately 29%, an unsatisfactorily low number that remains to be improved. We first reviewed the molecular epidemiology derived from a deep proteogenomic resource in Taiwan. The nuclear factor erythroid 2-related factor 2 (NRF2)antioxidant mechanism was discovered to mediate the oncogenesis and tumor progression of lung adenocarcinoma. Additionally, DNA replication, glycolysis and stress response are positively associated with tumor stages, while cell-to-cell communication, signaling, integrin, G protein coupled receptors, ion channels and adaptive immunity are negatively associated with tumor stages. Three patient subgroups were discovered based on the clustering analysis of protein abundance in tumors. The first subgroup is associated with more advanced cancer stages and visceral pleural invasion, as well as higher mutation burdens. The second subgroup is associated with *EGFR* L858R mutations. The third subgroup is associated with PI3K/AKT pathways and cell cycles. Both EGFR and PI3K/AKT signaling pathways have been shown to induce NRF2 activation and tumor cell proliferation. We also reviewed the clinical evidence of patient outcomes in Taiwan given various approved targeted therapies, such as EGFR-tyrosine kinase inhibitors and anaplastic lymphoma kinase (ALK)inhibitors, in accordance with the patients’ characteristics. Somatic mutations occurred in *EGFR, KRAS, HER2* and *BRAF* genes, and these mutations have been detected in 55.7%, 5.2%, 2.0% and 0.7% patients, respectively. The *EGFR* mutation is the most prevalent targetable mutation in Taiwan. *EML4-ALK* translocations have been found in 9.8% of patients with wild-type *EGFR*. The molecular profiling of advanced NSCLC is critical to optimal therapeutic decision-making. The patient characteristics, such as mutation profiles, protein expression profiles, drug-resistance profiles, molecular oncogenic mechanisms and patient subgroup systems together offer new strategies for personalized treatments and patient care.

## 1. Characteristics of Lung Cancer Patients in Taiwan

The lung is a vital organ responsible for the exchange of oxygen and carbon dioxides, and sustaining metabolism in humans. Lung cancer is the major cause of death for males and females with malignant tumors in Taiwan [1]. Only 15~20% of patients are diagnosed at early stages. Lung cancer comprises small cell lung cancer and non-small cell lung cancer (NSCLC), the predominant histology type, which is further categorized into adenocarcinoma, squamous cell carcinoma, large cell neuroendocrine carcinoma and others. The 5-year survival rate of Taiwanese patients with lung cancer has been reported to be approximately 28.6% in 2019. Of the ten most common malignancies in Taiwan, lung cancer has the lowest 5-year survival rate. The number of patients who died as a result of lung cancer increased from 5749 to 9701 between 1998 and 2019, suggesting that lung cancer is still a severe threat to the health and well-being of Taiwanese people [2]. The incidence of lung cancer in Taiwan has not decreased with time and the high mortality rate emphasizes the unmet need for optimizing treatment by leveraging a more personalized approach, including finding precise cancer subtypes, seeking driver oncogenes, overcoming drug resistance, and developing the optimal sequences of treatments [1].

## 2. Molecular Epidemiology and Pathophysiology of Lung Cancer in Taiwan

The distribution of patients with distinct molecular characteristics in a population, i.e., molecular epidemiology, is crucial for guiding the treatment policy to provide personalized treatments. NSCLC patients in Taiwan comprise higher percentages of nonsmokers than smokers, and females than males (Table 1). This is quite different from the demographics in western countries, such as the United States where there are more male patients and smokers (Table 1). Thus, molecular epidemiology of Taiwan warrants investigation. Recently, a comprehensive and deep proteogenomic resource of a cohort of treatment-naïve early-stage lung cancer patients in Taiwan was released, comprising high-quality molecular assessments of tumor and normal adjacent tissues (NAT) of surgical specimens, including gene expressions quantified by RNAseq and protein abundances quantified by mass spectrometry [3]. In this cohort, the majority of patients have adenocarcinoma. This resource facilitated the elucidation of lung cancer biology and molecular epidemiology in Taiwan.

Somatic mutation analysis of this cohort echoed a well-known fact that patients with the *epidermal growth factor receptor* (*EGFR*) mutations in the tumor account for 85% of the total patient population which comprises predominantly nonsmokers (83%) [3]. *RBM10* and *EGFR* L858 mutations are more common in females, whereas *KRAS* and *APC* mutations are frequently found in male patients. Additionally, *ATM* and *KRAS* are often found to have mutated in smokers.

Gross functional evaluations of increasing and decreasing proteins in this deep proteogenomic resource was performed with respect to the tumor stages. The increasing proteins are associated with DNA replication, glycolysis and stress response. The decreasing proteins pertain to cell-to-cell communication, signaling, integrin, G protein coupled receptors, ion channels and adaptive immunity. Based on the protein profiles of the tumor microenvironment, patients were clustered into three subgroups. The first subgroup accounts for 61% of the cohort. This subgroup is associated with more advanced cancer stages and visceral pleural invasion, as well as higher mutation burdens. Subgroup 2 accounts for 12% of the cohort and is associated with *EGFR* L858R mutations. Subgroup 3 accounts for 27% of the cohort and is associated with PI3K/AKT pathways and cell cycles, identified by protein phosphorylation analysis. Moderate RNA-to-protein correlations are found in this study, resulting in a slight disparity in the patient subgroups detected using RNA and protein alone [3].

**Figure 1:**
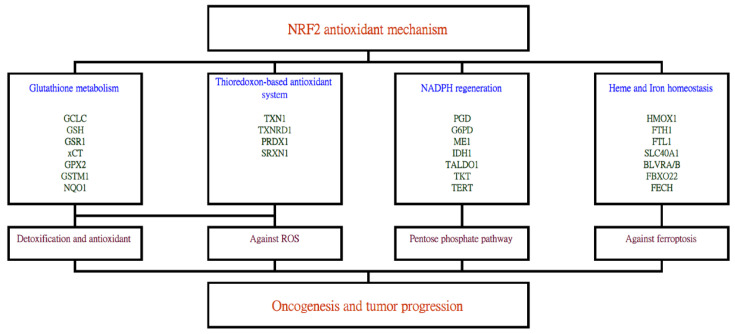
The stress-induced NRF2 antioxidant system can activate a series of downstream genes which ultimately leads toward lung cancer carcinogenesis and progression. Genes activated by the NRF2 transcription factor include those related to glutathione metabolism, thioredoxin-based antioxidant system, NADPH regeneration and heme and iron homeostasis. Subsequent effects, such as detoxification, reactive oxygen species removal, pentose phosphate pathway and inhibition of ferroptosis then follow.

An independent analysis of this proteogenomic resource further elucidated that the nuclear factor erythroid 2-related factor 2 (NRF2)/nuclear factor erythroid-derived 2-like 2 (NFE2L2) antioxidant mechanisms underlie the oncogenesis and tumor progression of lung adenocarcinoma in Taiwan, based on the higher expressions of NRF2 antioxidant genes in the tumor than in adjacent normal tissues [5]. Data suggested that the NRF2 antioxidant mechanism was the most prominent mechanism, among a total of 189 oncogenic mechanisms evaluated. This was a gross analysis performed regardless of the three subgroups mentioned above, with the goal of discovering the shared driving mechanism. To cope with the carcinogens and/or oxidative stress caused by the fast division of cells, NRF2 antioxidant mechanisms are induced. NRF2 is a transcription factor that can regulate the expression of other genes via binding with the antioxidant-response elements (ARE) of the genome and activating a series of downstream effects (Figure 1). These activities altogether mediate the metabolic reprogramming and increased antioxidant capability of the cancer cell.

Apart from the aforementioned oncogenic mechanisms derived from this proteogenomic resource, new insights can still be obtained using more advanced multi-omics analysis methods, such as similarity network fusing [6]. Furthermore, several important mechanisms are worth noting. Tissue fibrosis and inflammatory mechanisms have been found to be an important molecular signature associated with cancer stage and the poor prognosis of lung cancer [7]. This is a mechanism akin to wound healing. Patients with lung cancer often have metastasis to the brain. Thus, the cross-referencing of brain and lung cancer gene-expression signatures is warranted [8]. Additionally, eukaryotic initiation factors represent crucial members which can activate oncogenic pathways [9].

Like most cancers, lung adenocarcinoma is a heterogeneous collection of diseases. Partitioning a heterogeneous collection of diseases into relatively homogeneous subgroups allows for more precise estimation of patient outcomes and more precise subgroup-specific treatments [10]. The established oncogenic mechanisms, including *EGFR* mutation, *anaplastic lymphoma kinase* (*ALK*) fusion, *ROS1* fusion and *RET* fusion, only account for certain proportions of the entire patient population [4]. Hence, the search for new subtypes that better characterize the patient population is warranted. In research conducted in Japan, a country that is geographically close to Taiwan, two subtypes of lung adenocarcinoma were found using hierarchical clustering methods analyzing the miRNA profiles in the tumor tissues. The two subtypes are driven by the dysregulation of miRNA miR-30d and miR-195, respectively. The first subtype represents less differentiated tissue and implies poorer survival. The second subtype represents well-differentiated tissue and implies better survival [11]. In another study utilizing The Cancer Genome Atlas (TCGA) data, three subtypes (high, medium and low immunity) were identified [12]. The high-immunity group has a better response to immunotherapy and chemotherapy.

## 3. Translational Oncology Studies of Lung Cancer in Taiwan

### 3.1. EGFR

The molecular profiling of advanced NSCLC is critical to optimal therapeutic decision-making. EGFR mutation is the most prevalent targetable mutation in Taiwan and could be detected in approximately 56% of patients with pulmonary adenocarcinoma [1,13]. The first prospective study in Taiwan to identify the incidence of five driver oncogenes, including *EGFR*, *Kirsten rat sarcoma viral oncogene homolog (KRAS)*, *B-Raf proto-oncogene serine/threonine kinase (BRAF)*, *human epidermal growth factor receptor 2 (HER2)* and *echinoderm microtubule-associated protein-like 4-anaplastic lymphoma kinase (EML4-ALK)* fusion mutations in 1772 patients with treatment-naïve pulmonary adenocarcinoma showed that EGFR, KRAS, HER2 and BRAF mutations were detected in 987 (55.7%), 93 (5.2%), 36 (2.0%) and 12 (0.7%) patients, respectively [14]. Most of the above-mentioned mutations were mutually exclusive. In addition, 29 of 295 EGFR-wild type patients (9.8%) were found to have EML4-ALK translocation. EGFR mutations were more frequent in female patients and never-smokers [14]. EGFR exon 19 deletions (44.8%) and exon 21 L858R (47.9%) were shown to be the main subtypes of EGFR mutations. Fewer Taiwanese patients aged ≤ 50 years have been reported to have EGFR mutations, and the subtypes of EGFR mutation were more uncommon. Age ≤ 50 years was associated with inferior efficacy of EGFR-tyrosine kinase inhibitors (TKI) [15,16,17,18]. Mutant plasma EGFR (pEGFR) was reported to be a poor prognostic factor in EGFR-mutant patients [19]. A retrospective study showed that multiple primary malignant tumors occurred more frequently in patients with lung cancer harboring classic EGFR mutations, particularly those with exon 19 deletions [20]. Patients with adenocarcinoma of the lung who also had scar cancer or old pulmonary tuberculosis lesions were shown to have a higher probability of harboring EGFR mutations, particularly exon 19 deletions [21]. EGFR-tyrosine kinase inhibitors (EGFR-TKIs), including gefitinib [22], erlotinib [23], afatinib [24], dacomitinib [25] and osimertinib [26] are treatment options as a first-line therapy for advanced NSCLC patients with EGFR mutations [27,28,29,30]. The survival benefit of EGFR-TKIs has also been reported in nonagenarian patients with advanced EGFR-mutant NSCLC [31].

The approved first-line treatment for advanced EGFR-mutant NSCLC in Taiwan includes gefitinib, erlotinib, afatinib, dacomitinib, and osimertinib. However, the third-generation EGFR-TKI, osimertinib, is only reimbursed by the National Health Insurance (NHI) as a first-line treatment for advanced EGFR exon 19 deletion-positive NSCLC with central nervous system metastasis. Therefore, many patients still received second-generation EGFR-TKIs, including afatinib, as the first-line treatment [1]. Previous reports showed that afatinib as a first-line treatment provided better survival outcomes for advanced EGFR-mutant lung adenocarcinoma than gefitinib and erlotinib [32,33,34]. Afatinib is also effective for pulmonary adenocarcinomas with complex EGFR mutations, especially those with uncommon mutations [33]. Erlotinib and afatinib revealed better treatment efficacy in patients with initial brain metastases than gefitinib [33]. In a retrospective study, alternative treatment with Chinese herbal medicine (CHM) during first-line EGFR-TKI showed a tolerable toxicity profile and a tendency toward better progression-free survival (PFS)and overall survival (OS) than those who did not receive CHM [35]. Multiple studies have reported that not only erlotinib plus bevacizumab, but also afatinib combined with bevacizumab, demonstrated favorable efficacy for the first-line treatment of advanced pulmonary adenocarcinoma harboring EGFR mutations [36,37,38]. A multicenter observational study reported that the first-line afatinib plus bevacizumab demonstrated an ORR of 87.7%, a median PFS of 23.9 months, and a median OS of 45.9 months. Further prospective studies are warranted to confirm the clinical efficacy [37,38]. In addition, immunotherapy plus chemotherapy tended to be more effective than immunotherapy alone in previously TKI-treated NSCLC harboring EGFR mutations [39]. A study showed that the level of immunosuppressive cells and immune checkpoint proteins increases during the EGFR-TKI resistance period, implicating a critical mechanism for tumor progression [40]. The decreased infiltration levels of CD8^+^ T cells after the development of EGFR-TKI resistance have also been reported [41]. Therefore, the combination of EGFR-TKI and immunotherapy has been investigated in multiple studies, and current data showed controversial findings [42].

Among patients with EGFR-mutant advanced pulmonary adenocarcinoma who had acquired resistance to first-line TKIs, pemetrexed-based doublet chemotherapy has been shown to be the preferred chemotherapy regimen until a more efficient therapeutic option is identified. Moreover, PFS and OS were longer in patients who underwent combination chemotherapy than single-agent chemotherapy [42]. A study that enrolled 330 patients with advanced EGFR-mutant pulmonary adenocarcinoma showed that elderly patients aged 70 years or older with disease progression after first-line TKI could receive chemotherapy and had a response rate similar to that of younger patients [43]. The retreatment of first/second-generation EGFR-TKIs after initial TKI followed by chemotherapy has been reported to be effective in prolonging survival, and a useful option for subsequent treatment, especially in patients with longer drug holidays and female patients with exon 21 mutation [44,45,46,47]. Patients with EGFR-mutant pulmonary adenocarcinoma have been found to have a higher proportion of synchronous liver metastasis (LM) than those with EGFR wild-type tumor, and patients with an adequate performance status and ≤5 LM nodules could be considered for systemic therapy combined with radiofrequency ablation when LM develops [48].

Acquired resistance to first/second-generation EGFR-TKI inevitably occurs regardless of which EGFR-TKI is used. The most common molecular resistance mechanism to first/second-generation TKIs is the EGFR T790M mutation in exon 20 which could be treated with osimertinib and accounts for about 50–60% of patients [1,49]. The T790M acquisition rate was around 46.3–55.1% in Taiwan [50,51,52,53]. Higher frequency of T790M acquisition in patients with exon 19 deletion, L858R, longer treatment duration of previous EGFR-TKIs, previous gefitinib treatment, initial liver metastasis, and rebiopsy at metastatic sites has been reported. The longer duration between the first and second rebiopsy among patients with the T790M mutation in the second biopsy has been found compared to those without T790M [50,51,52,53,54,55]. The presence of circulating EGFR cell-free DNA, including the exon 19 deletion, L858R mutation, or T790M mutation showed different influences on OS in patients with advanced EGFR-mutant pulmonary adenocarcinoma who have progressed on first-line EGFR-TKIs [55]. Complex EGFR mutations with T790M and a shorter treatment duration of previous EGFR-TKIs have been associated with shorter PFS and OS in patients with advanced NSCLC treated with osimertinib [56]. Potential resistance mechanisms, including Cys797Ser, cMET amplification, BRAF mutation, KRAS mutation, and small-cell transformation, developed in patients who have progressive disease following osimertinib [57,58]. A retrospective study showed that continuous osimertinib in combination with other therapies was related to improved OS in patients who have progressed on osimertinib [59].

After treatment with first/second-generation EGFR-TKIs, patients with EGFR mutations were reported to have a higher frequency of developing brain metastasis than those without [60]. The optimal brain surveillance strategy during EGFR-TKI treatment remains unclear. One retrospective study revealed that the strategy of regular brain magnetic resonance imaging (MRI) every 3–6 months did not demonstrate a significant impact on the survival outcome, irrespective of initial brain metastasis [61]. A single-center study showed a significantly higher proportion of patients with EGFR-mutant NSCLC died from central nervous system (CNS) metastases compared to those with EGFR wild-type tumors [62]. The addition of stereotactic radiosurgery (SRS) to EGFR-TKI therapy has been reported to improve intracranial tumor control and provide a potential benefit of preventing neurological deficits and seizures. [63] The combined use of Gamma Knife radiosurgery and EGFR-TKI was shown to have the most significant effect on prolonging survival time after SRS in patients with EGFR-mutant lung cancer and brain metastasis [64]. A prospective study showed that the supernatant of cerebrospinal fluid (CSF) in patients with EGFR-mutant NSCLC and leptomeningeal metastasis (LM) is a valuable specimen for EGFR mutation testing, demonstrating that the detection rates of EGFR mutations and T790M in cell-free DNA (cfDNA) of CSF were 68.8%, and 14.6%, respectively. [65] The best way to use CSF for diagnosis, tracking tumor heterogeneity, or treatment guidance requires further investigation [66]. CSF diversion, including ventricular peritoneal shunt, and lumbar peritoneal shunt has been reported to be a useful and safe management for LM and hydrocephalus [67].

Stage III NSCLC includes a very heterogeneous group of patients who require different treatment strategies. An investigation that enrolled 92 cases with unresectable stage III pulmonary adenocarcinoma reported that patients with EGFR-mutant adenocarcinoma who underwent upfront EGFR-TKIs had significantly superior PFS and OS than those who underwent upfront concurrent chemoradiation. An observational study of stage III NSCLC in Taiwan showed that upfront chemoradiotherapy with subsequent EGFR-TKI provided a longer survival time compared to upfront EGFR-TKI in patients with a stage III unresectable EGFR-mutant NSCLC [68]. The above-mentioned information suggests that further randomized studies are necessary to validate these findings [69].

### 3.2. ALK

About 10% of cases with EGFR wild-type pulmonary adenocarcinoma are positive for EML4-ALK rearrangements in Taiwan [1]. ALK translocation is associated with ALK expression and mutually exclusive with EGFR mutation in Taiwanese patients with NSCLC [70]. A multicenter prospective study showed that 29 of 295 (9.8%) EGFR-wild type patients with lung adenocarcinoma were positive for EML4-ALK translocation, which occurred more frequently in patients younger than 65 years [14,16]. Another study reported that 124 of 1255 (9.9%) patients with EGFR-wild type NSCLC were ALK-positive [71]. Among them, the rates of programmed death-receptor ligand-1 (PD-L1) tumor proportion score (TPS) ≥1% and ≥50% were 50% and 16%, respectively, and no significant relation between PD-L1 expression and ALK fusion variants was found. The characteristics of ALK-positive NSCLC in Taiwan are different from those in western countries, such as the United States (Table 2) [71,72,73,74,75]. In Taiwan, positive PD-L1 expression (≥1%) was related to unfavorable PFS in patients with ALK-positive pulmonary adenocarcinoma receiving crizotinib [72,76]. In addition, neither ALK variants nor the Bcl-2-like 11 (BIM) polymorphism was related to the PFS or OS in ALK-positive NSCLC receiving crizotinib [77]. ALK mutations were found more frequently in cases with age < 50 years, female gender, non-smokers, and the histology type of adenocarcinoma. Age ≤  50 years and non-smokers were independent predictors for ALK mutation. The ALK mutation rate in the never/light smokers aged less than 50 years with EGFR-wild type NSCLC could be as high as 23% [71].

ALK inhibitors, including crizotinib, alectinib, ceritinib, brigatinib, and lorlatinib have been approved in Taiwan, but only crizotinib, alectinib, and ceritinib are reimbursed by the NHI as the first-line treatment for advanced ALK-positive NSCLC [1].

One prospective multicenter study to investigate the resistance mechanisms in patients with advanced NSCLC who progressed after ALK inhibitors demonstrated heterogeneous mechanisms of resistance, including the loss of ALK mutations in less than one-third of cases, in addition to various compound ALK mutations, and bypass mutations [78]. Therefore, rebiopsy to discover the resistant mutations might be considered to determine the optimal treatment for ALK-inhibitor-resistant NSCLC [79]. In retrospective studies of ALK-positive advanced NSCLC, ceritinib demonstrated a longer median PFS compared to crizotinib for first-line treatment [80], and a second-generation ALK inhibitor, alectinib demonstrated a lower incidence of CNS progression and a tendency toward superior PFS compared to ceretinib in specific patients who had progressed on crizotinib [81].

### 3.3. Rare Mutation Other Than EGFR and ALK

A multicenter prospective study in Taiwan enrolled 1772 patients and demonstrated that BRAF, HER2 and KRAS mutations were identified in 12 (0.7%), 36 (2.0%) and 93 (5.2%) patients, respectively. KRAS mutations were more frequently detected in male patients and smokers. Most of the genetic alterations were mutually exclusive, except for co-occurring mutations in seven (0.4%) patients, including one with KRAS and BRAF mutations, three with KRAS and EGFR mutations, and three with HER2 and EGFR mutations [14]. The incidence of mesenchymal-epithelial transition (MET) exon 14 skipping mutation has been reported to be 3.3% for lung cancer and 4% for pulmonary adenocarcinoma. Patients harboring MET exon 14 skipping mutation without anti-MET therapy had a similar OS compared to those without a major driver gene mutation [82].

In Taiwan, dabrafenib plus trametinib is approved for the treatment of advanced BRAF V600E-positive NSCLC but is not reimbursed by the NHI [1] Crizotinib and entrectinib are approved and reimbursed for the treatment of advanced ROS1-positive NSCLC. Capmatinib and tepotinib have received approval for the treatment of MET exon 14 skipping mutation but are not reimbursed. Larotrectinib and entrectinib have been approved for metastatic NTRK fusion-positive NSCLC but are not reimbursed by the NHI. No drug has received approval for the treatment of HER2 mutation-positive and KRAS-mutated NSCLC as of May 2022 in Taiwan [1].

Whole-exome or targeted gene sequencing was used in one study and showed that KRAS and TP53 mutations were identified in 5.56% and 25% of Taiwanese patients, respectively. The other mutations, including ARID1A, ARID2, CDK12, CHEK2, GNAS, H3F3A, KDM6A, KMT2C, NOTCH1, RB1, RBM10, RUNX1, SETD2, SF3B1, SMARCA4, THRAP3, TP53, and ZMYM2 were also found. The mutation profiles in Taiwanese patients may have a critical influence on the determination of targeted therapies. [83] One study demonstrated that the inactivation of TP53/RB1 function may be associated with the histogenesis of synchronous/metachronous SCLC/NSCLC. In addition, the SCLC component may derive from the adenocarcinoma component through the activation of achaete-scute family bHLH transcription factor 1 (ASCL1) and PI3K/AKT1 signal transduction pathways [84]. The PIK3CA mutation rate was reported to be 1.8% (14/760) in lung cancer without EGFR-TKI treatment. No significant different treatment response or PFS of EGFR-TKI was found between patients with PIK3CA mutation and those without. The PIK3CA mutation rate in patients with pulmonary adenocarcinoma who have progressed on EGFR TKI was not higher than that in patients without EGFR-TKI treatment [85].

The KIF5B-MET fusion variant has been detected in one person (0.5%) out of 206 pulmonary adenocarcinomas. Among 28 patients with pulmonary sarcomatoid carcinoma, one case with a KIF5B-MET fusion variant (3.6%) was identified and had rapid progression of tumor invasion. This patient passed away about 1 month after diagnosis with lung cancer [86].

In the aforementioned cohort with early-stage, predominantly female, and non-smoking pulmonary adenocarcinoma, the mutational signature analysis showed a high prevalence of APOBEC mutational signature in younger females. Moreover, in older females, overrepresentation of environmental carcinogen-like mutational signatures was found [3].

A study regarding mitochondria genome in stage I pulmonary adenocarcinoma showed that patients with somatic mutations in the D-loop region had longer relapse-free survival (RFS) than those without, whereas somatic mutations in mitochondrial Complex IV and Complex V genes were related to shorter RFS [87]. In pulmonary adenocarcinoma, the NGS study also showed six potential oncogenic genes, including AGR2, SPDEF, CDKN2A, CLDN3, SFN, and PHLDA2, and seven tumor suppressor genes [88].

### 3.4. Small Cell Lung Cancer

The most aggressive type of lung cancer in Taiwan is small-cell lung cancer (SCLC) characterized by rapid growth and early metastasis [89,90]. A large observational population-based cohort study of SCLC in Taiwan revealed that more than 80% of the patients were ES-SCLC at diagnosis [90]. SCLC is highly responsive to chemotherapeutic drugs, and no targeted therapies have been approved for the treatment of SCLC to date. Chemotherapy is the mainstay of initial treatment for ES-SCLC, prolonging survival in comparison with best supportive care [91]. The addition of immunotherapy to doublet chemotherapy provides further survival benefits [92,93,94]. The median survival times for Taiwanese patients with LS- and ES-SCLC have been reported to be 16.92 and 8.71 months, respectively [90]. The preferred and superior first-line chemotherapy regimen was etoposide plus platinum. The preferred second-line treatment for relapsed SCLC was topotecan which has demonstrated limited efficacy [90].

A study that enrolled 76 patients with SCLC revealed two (2.6%) cases with EGFR mutations in which both were exon 19 deletions. [95] Another study showed that one case (1.6%) with EGFR exon 21 L858R was detected among 63 patients with SCLC [96]. SCLC with EGFR mutations may be attributed to the combined component of adenocarcinoma [95]. An investigation of combined SCLC and NSCLC demonstrated that the inactivation of TP53/RB1 function is related to the histogenesis of de novo combined SCLC/NSCLC and pulmonary adenocarcinoma with SCLC transformation after EGFR-TKI treatment. Furthermore, the activation of the PI3K/AKT1 and ASCL1 signal transduction pathways was shown to be associated with the transformation of adenocarcinoma to SCLC [84].

## 4. Discussion

Patient demographics of lung cancer in Taiwan are different from those in western countries. With a high percentage of young female patients and high proportions of non-smokers in Taiwan, the etiology of the tumor occurrence is largely elusive. Air pollution has been nominated as one culprit. A lack of definitive etiology makes it difficult at this time to avoid the carcinogens in people’s daily life. Low-dose computed tomography (CT) is a valuable tool for detecting lung cancer at the early stages. In 2013, the results of the US National Lung Cancer Screening Trial indicated that low-dose CT is useful in high-risk lung cancer populations. In 2014, the American College of Radiology published the Lung-RADS guidelines for low-dose CT lung cancer screening. In Taiwan, the low-dose CT screen of lung nodules has become more and more popular to be included in regular health examination packages. These would facilitate the detection of lung cancer at earlier stages when the patients have better prognosis given treatments.

The treatment of advanced lung cancer is guided by the molecular characteristics of the tumor. Current treatment guidelines specify the following procedure for the systemic treatments of patients [1,97,98,99,100,101,102,103,104,105,106]. For NSCLC, activation of EGFR, ALK, ROS-1, and other targetable genes in the tumor are evaluated. Antagonistic agents are given if any of the oncogenic activations mentioned above are found [22,23,24,25,97,98,99,100,101,102,103,104,105].

With this treatment algorithm in place, the 5-year survival rate of Taiwanese patients with lung cancer is approximately 29%, an unsatisfactorily low number that remains to be improved. One of the reasons is that certain patients lack EGFR, ALK, ROS-1 and other targetable mutations to support a rational treatment of these pathogenic mechanisms. Hence, it is warranted to investigate under-recognized patient subgroups and their corresponding mechanisms, so as to design corresponding targeted strategies. This could be achieved using both the top-down and bottom-up approaches. Top-down approaches are exemplified by the discovery of NRF2 activations, which were detected in all lung cancer tissues regardless of the status of other biomarkers, such as EGFR, ALK, ROS-1 and other genetic alterations. The detected mechanism thus represents an underlying mechanism with various degrees of importance in different patient subgroups. One other example is the aforementioned identification of three patient subgroups, including the PI3K/AKT patient subgroup, which may encourage the development of PI3K/AKT inhibitors for lung cancer.

Interestingly, both EGFR and PI3K/AKT signaling pathways have been shown to induce NRF2 activation and tumor cell proliferation [107,108]. The NRF2-mediated cell proliferation is dually regulated by EGFR and an NRF2-repressor protein, KEAP1 [107]. NRF2 is constitutively activated by EGFR signaling in the presence of EGFR activation mutations [108]. Since NRF2 is a downstream molecule of EGFR, oxidative stress reduces the anticancer effect of EGFR-TKI [107]. Additionally, the inhibition of the PI3K pathway markedly attenuated the expression of NRF2 downstream genes [108]. Constitutively active AKT mutants stimulated NRF2 activation. Thus, the NRF2 antioxidant mechanism represents a source of counteracting strategies to deter cancer progression, particularly in cases when drug resistance has been acquired in upstream molecules.

We can also perform bottom-up investigations, by checking all the decision points in the current treatment roadmaps to see if better molecular markers can be used to indicate a better treatment for an individual patient. This way, precision medicine can be achieved, and the survival rates of the patients could be improved.

## 5. Conclusions

Lung cancers are responsible for great healthcare burdens in Taiwan, and the medical institutes here have been giving state-of-the-art treatments to the patients. To learn from these valuable treatment experiences, we reviewed the genomic landscape of lung cancer and the treatment efficacies of various approved targeted therapies in patients with different mutation profiles. The somatic mutations in *EGFR*, *KRAS*, *HER2* and *BRAF* genes have been detected in 55.7%, 5.2%, 2.0% and 0.7% patients, respectively. *EGFR* mutation is the most prevalent targetable mutation in Taiwan. *EML4-ALK* translocations have been found in 9.8% of patients with wild-type *EGFR*. The molecular profiling of advanced NSCLC is of prime importance to optimal therapeutic decision-making. To further elucidate the molecular epidemiology and underlying oncogenic mechanisms, we reviewed a deep proteogenomic resource in Taiwan. The discovery of the NRF2 oncogenic mechanism as an important driving mechanism in Taiwan and the discovery of the patient subgroup system offers new angles for deriving future counteracting strategies against specific lung cancer mechanisms. Further studies are warranted to discover more genetic alterations, protein expression profiles, drug-resistance profiles, and signal transduction pathways that are essential to the development of lung cancer. Integration of molecular and clinical data would be critical to better understand lung cancer in Taiwan and improve the outcome of this fatal disease.

## Figures and Tables

**Table 1:** Distinct demographics of NSCLC patients in Taiwan compared with those in United States.

Demographics	Taiwan [3]	United States [4]
**Gender**		
Female	60.67%	35.51%
Male	39.33%	64.49%
**Smoking History**		
Non-smoking	86.52%	44.00%
Smoking	13.48%	56.00%

**Table 2:** Characteristics of ALK-positive NSCLC in Taiwan compared with those in the United States.

	Taiwan [71,72]	United States [73,74,75]
**Age, Median (Range), Years**	**55.1 (32–89)**	**52 (29–76)**
Male gender (%)	46	58
**Smoking History (%)**		
Non-smoking	65	74
Smoking	35	26
**Metastatic Site (%)**		
Brain	25	33.3
Liver	17.3	16.7
Bone	38.5	33.3
**PD-L1 Expression (%)**		
PD-L1 TPS 0%	50%	37%
PD-L1 TPS 1–49%	34%	37%
PD-L1 TPS ≥ 50%	16%	26%
